# Properties and Microstructure Evaluation of Laser-Welded TP347—TP904L High-Alloy, Stainless Steels Joints, Modified with 309L Filler

**DOI:** 10.3390/ma18245633

**Published:** 2025-12-15

**Authors:** Hubert Danielewski, Piotr Kurp, Andrzej Skrzypczyk, Jindřich Kozák, Pavel Konopík, Jianhua Yao, Qunli Zhang, Sylwia Rzepa

**Affiliations:** 1 Laser Research Centre, Faculty of Mechatronics and Mechanical Engineering, Kielce University of Technology, Al. Tysiąclecia P.P. 7, 25-314 Kielce, Poland; pkurp@tu.kielce.pl (P.K.); andrzej.skrzypczyk@gmail.com (A.S.); 2 Department of Mechanical Technology, Faculty of Mechanical Engineering, VŠB-Technical University of Ostrava, 17 listopadu 15, 708 33 Ostrava, Czech Republic; jindrich.kozak@vsb.cz; 3 COMTES FHT a.s., Průmyslová 995, 334 41 Dobřany, Czech Republic; pavel.konopik@comtesfht.cz; 4 Institute of Laser Advanced Manufacturing, Zhejiang University of Technology, No. 288 Liuhe Road, Hangzhou 310023, China; laser@zjut.edu.cn (J.Y.); zql@zjut.edu.cn (Q.Z.); 5 Academic Centre for Materials and Nanotechnology, AGH University of Krakow, Mickiewicza 30, 30-059 Kraków, Poland; srzepa@agh.edu.pl

**Keywords:** laser welding, filler material, high alloy, stainless steels, TP347 and TP904L steels, weld microstructure analysis, mechanical properties analysis

## Abstract

This study presents the results of laser beam welding of dissimilar high-alloy super stainless steels. Differences in their thermal and mechanical properties pose significant challenges in manufacturing processes. The present work demonstrates the potential advantages of using 309L filler material in laser welding of high-alloy materials with different properties. The research focuses on a comparative evaluation of the effects of 309L filler metal on the TP904L—TP347 joint in terms of joint strength and microstructure. The analysis of the joints provides insight into the role of the filler metal in improving joint properties. The obtained results show that both welds exhibit a similar microstructure composed of pillar, cellular, and equiaxed dendrites; however, they differ in dendrite growth orientation, calculated ferrite number (FN), the G/R ratio, and dendrite arm spacing, indicating a lower thermal gradient in the joint welded with filler metal. The results also reveal the presence of precipitates in the welds near the TP904L steel fusion line, most likely Cr_23_C_6_ type. Mechanical properties evaluation, based on standard and miniaturized tensile tests as well as hardness measurement, shows that the use of 309L filler metal improves both the joint strength and ductility, although it does not significantly affect the material hardness.

## 1. Introduction

Laser beam welding (LBW) is a high-precision joining method that provides minimal thermal distortion, and it is widely used in various industrial sectors, including aerospace, automotive, and energetic installations [1,2,3,4]. According to numerous studies, one of the most challenging tasks in LBW is the welding of dissimilar high-alloy materials. Such materials often differ significantly in their thermal properties, including melting points, thermal conductivities, and thermal expansion coefficients [5,6]. These disparities can lead to welding defects, such as porosity, cracking, and undesirable precipitation formation, which may reduce the mechanical properties and operational lifetime of welded components [7]. Among the aforementioned industries, the energy sector, including nuclear installations, superheated steam systems, and biomass boilers, requires the highest joint quality level to ensure safe and long-term operation.

Currently, a trend toward replacing martensitic steels with new high-alloy austenitic steels has emerged in critical components of energy systems [8,9]. The properties of these materials, such as high-temperature resistance, corrosion resistance, and creep resistance, must be preserved during manufacturing and service [10]. Studies presented by various authors [11,12] have shown that LBW can meet these requirements. Moreover, when further enhancement of specific joints’ selected properties, such as corrosion resistance or weld formability, is required, laser welding with filler material can provide those advantages [13,14].

Manufacturing energy-sector components from dissimilar materials, when supported by an appropriate joining technology, offers significant advantages when specific material properties are required. Among steels commonly used in this sector, super-austenitic stainless steels TP904L, known for its excellent corrosion resistance, and TP347, niobium-stabilized stainless steel with high-temperature resistance and oxidation resistance, both exhibit considerable application potential [15]. Although both materials offer high-temperature resistance, TP347 provides better corrosion and creep resistance at elevated temperatures, whereas TP904L offers superior corrosion resistance in aggressive environments [16,17]. Despite their generally good weldability, joining these steels presents specific challenges associated with their thermal and metallurgical characteristics. Differences in crystallization behaviour, including the suppression of ferrite formation due to the high Ni content in TP904L, increase the risk of hot cracking. Furthermore, the elevated Mo content may promote the formation of eutectic structure and σ-phase. In addition, the absence of Nb in TP904L composition—which normally stabilizes carbide formation—can lead to the development of precipitations such as Cr_23_C_6_ during solidification or subsequent cooling [18].

The purpose of the presented study is to investigate the possibilities of using these materials together with laser beam welding techniques to manufacture components for energy installations. The research includes a comparative analysis of the joint properties in cases where the materials are welded together without and with the use of filler metal. The literature study indicates a lack of publications analyzing TP347—TP904L joints, particularly those presenting improvement of joint properties achieved through LBW with filler metal.

In order to achieve a high-quality TP347—TP904L joint, an approach involving the modification of microstructure and properties through the use of filler material is proposed. Laser welding with filler metal can be used to improve mechanical properties, reduce the thermal gradient (thus minimizing the risk of hot cracking), and improve the overall quality of the joint [19]. Due to the high Cr and Ni content of both base materials, an austenitic filler wire of grade 309L (diameter 1 mm), characterized by high Cr, moderate Ni, and low Mo contents, was selected. Filler metal with such a composition promotes ferrite formation, reduces the risk of hot cracking, increases mechanical properties, and maintains high corrosion resistance. The microstructure of welded joints in austenitic steels depends on the crystallization mechanism and subsequent solid-state transformations. For steels with a higher chromium-to-nickel equivalent ratio, primary crystallization proceeds with the precipitation of δ-ferrite, whereas for steels with a lower ratio, austenite precipitates first. Based on the WRC diagram and calculated Cr_eq and Ni_eq values, the ferrite content and potential formation of brittle σ-phase can be predicted. By determining the ferrite number (FN) and using the WRC-1992 diagram, the tendency of the joint crystallization cracking can also be assessed [20].

The presented studies provide an answer as to how the use of 309L filler metal affects the mechanical strength, hardness, microstructure, and defect formation in the analyzed joint and its characteristic areas. Based on tensile strength testing, hardness measurements, optical microscopy, scanning electron microscope (SEM), and EDS analysis, the properties of super-austenitic steel joints welded using the LBW technique are discussed [21].

## 2. Materials and Methods

In the presented study, two different high-alloy stainless steels were used: TP904L and TP347. The chemical compositions of these materials, based on the inspection certificate, are presented in Table 1. TP904L steel was selected for its excellent corrosion resistance, whereas TP347 steel was chosen for its high-temperature strength and corrosion resistance. During the welding of TP904L, precipitates in the form of Cr_23_C_6_ are often observed; however, post-weld heat treatment can effectively prevent their formation [22,23]. Meanwhile, TP347 steel, due to carbon stabilization with niobium, does not exhibit problems related to carbide precipitation.

As an additional material, filler wire in grade 309L is used (Table 2) [24,25,26,27]. This filler was selected based on its chemical composition, compatibility with the base materials, superior mechanical properties, high-temperature service, good weldability, and preliminary research. In particular, for TP904L steel, which exhibits a tendency to hot cracking due to its fully austenitic solidification mode, the use of 309L filler helps to mitigate this risk [28].

The materials were used in the form of standardized sheets designated for welding process qualification, with dimensions of 300 × 150 mm and a thickness of 4 mm. Samples were cut using laser nitrogen cutting with elevated working gas pressure to ensure high-quality edges. For welding with filler materials, the top edges of sheets were chamfered to a depth of 1 mm at an angle of 15°. The LBW process was carried out using a Trumpf TruFlow 6000 CO_2_ laser integrated with a TruLaserCell 1005 machine (Trumpf Group, Ditzingen, Germany). The laser operated at a wavelength of 10.6 µm, with circular polarization, a focal point diameter of 0.3 mm, and a focal length of 270 mm. The laser output power, welding speed, filler wire feed rate, and chamfer depth and angle were optimized based on preliminary research to ensure complete joint penetration and a high level of weld quality. The welded sheets were secured in a specialized fixture to prevent displacement, with upper screws fixing and one side spring clamping. The parameters used in the welding process are presented in Table 3.

To compare the results of laser welding performed with and without filler metal, the process was carried out using the same laser power and welding speed. The main differences between the two welding procedures concerned the use of a filler metal and the resulting adjustments, such as the focal point position. In welding without filler, the focal point was placed on the surface of the fitted sheets, whereas for welding with filler, it was positioned on the surface of the wire surface, which was placed in the chamfered groove [29].

For metallographic analysis, specimens were extracted using a wire electric discharge machine (WEDM) (Industrial Automation Plant B.P, Kutno, Poland). The cross-section surfaces of the extracted specimens were ground and polished using a Buehler MetaServ 250 grinder (ITW Test & Measurement GmbH, Meisenweg, Germany), and then electrolytically etched with a 10% oxalic acid solution. SEM analysis was performed using a JEOL 7100F scanning electron microscope (JEOL Ltd., Tokyo, Japan) operated under high-vacuum conditions. Micrographs were acquired at an accelerating voltage of 15.0 kV with a working distance (WD) of 10 mm. Energy-Dispersive X-ray Spectroscopy (EDS) analyses were conducted using an X-Max detector (Oxford Instruments, Abingdon, UK) with a sensor area of 20 mm^2^ and an energy resolution of 127 eV. Optical microstructure analysis was carried out using a confocal digital microscope, Hirox KH-8700 (Hirox Co Ltd., Tokyo, Japan).

**Figure 1:**
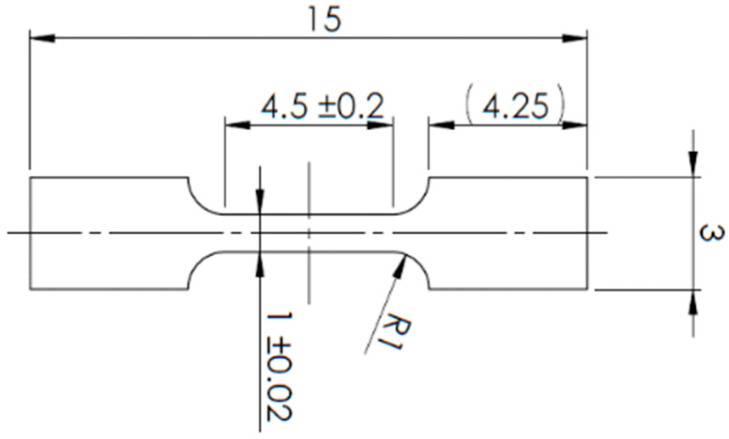
Schematic illustration of the miniaturized tensile test specimen geometry.

Miniaturized tensile tests were performed using a TiraTest universal testing machine (TIRA GmbH, Schalkau, Germany) with a loading capacity of 10 kN. Specimens were extracted from characteristic regions of the joints using WEDM, according to the schematics shown in Figure 1 and Figure 3. Deformation was recorded using a Mercury RT virtual extensometer (SOBRIETY s.r.o., Kuřim, Czech Republic) calibrated in 2D mode with a single camera. The initial gauge length of the virtual extensometer for sub-sized specimens was set to 4 mm. All the specimens were tested under quasi-static conditions at a strain rate of 0.00025 s^−1^ (0.07 mm/min) at room temperature.

**Figure 2:**
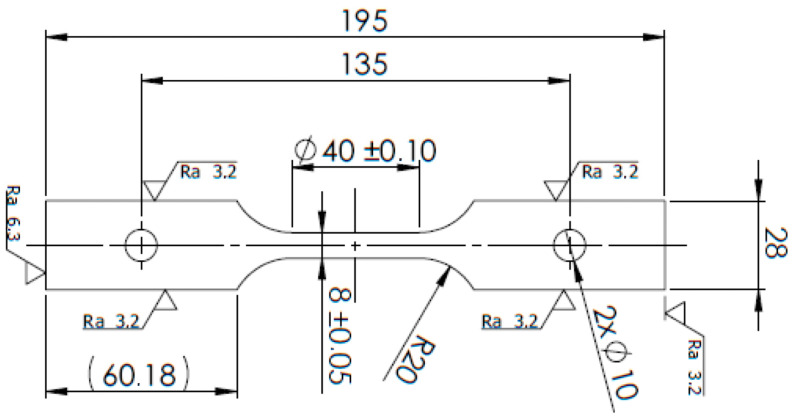
Schematic illustration of the standard tensile test specimen geometry.

**Figure 3:**
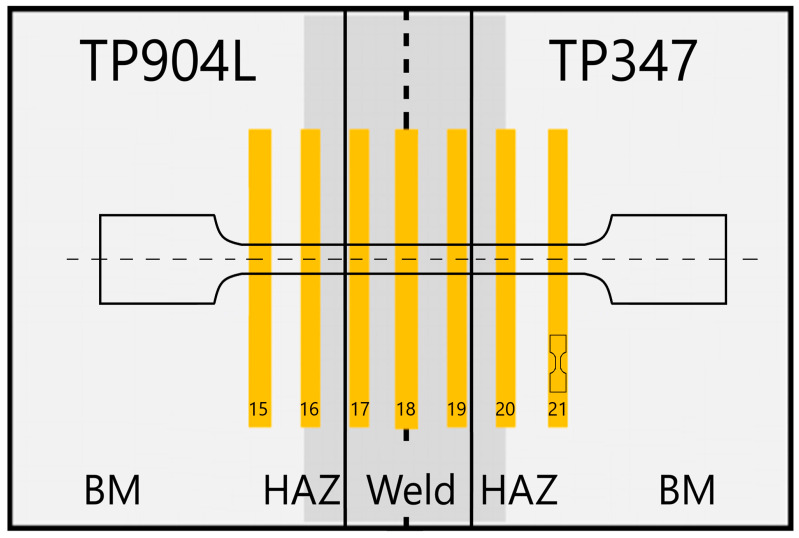
Extraction schema of miniaturized and standard tensile test specimens from the obtained joints.

The standard tensile tests of the obtained butt joints were performed using an INOVA universal hydraulic testing machine (Inova GmbH, Bad Schwalbach, Germany) with a loading capacity of 200 kN. Specimens were extracted using WEDM, according to the schematics shown in Figure 2 and Figure 3. Deformation was measured using a 12 MPx Aramis system (GOM) (Carl Zeiss AG, Oberkochen, Germany) with a single camera in 2D mode. A stochastic pattern was applied to the specimens for the optical displacement monitoring. The initial gauge length of the virtual extensometer (L_0_) was set to 50 mm.

Hardness measurements were carried out using the Innovatest Nexus 4303 machine (INNOVATEST Europe BV, Maastricht, The Netherlands), following the Vickers method (HV0.5).

## 3. Results

### 3.1. Global Observation

**Figure 4:**
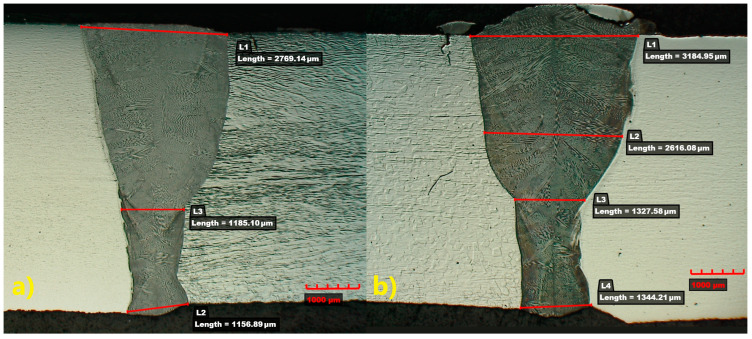
Macroscopic view in cross-section of obtained joints: (**a**) welded without filler material, (**b**) welded with 309L filler.

The obtained welded joint exhibits comparable geometries (Figure 4). The root of the weld in specimen (b) was slightly wider, measuring 1.34 mm, whereas in specimen (a), the weld width was 1.16 mm. In both cases, full material penetration was achieved, resulting in a weld depth of 4 mm.

Greater differences were observed in the upper regions of the welds. The joint welded with filler material exhibited a double-U profile (Figure 4b), whereas the joint welded without filler displayed a single-U profile (Figure 4a). The macrostructure analysis indicates proper weld build in both cases, with a slightly convex weld face, complete joint penetration, and no visible defects. The crack observed in Figure 1 is attributed to the preparation of the metallographic specimen.

The measured weld face width for specimen (a) was 2.77 mm, whereas for specimen (b) it was 3.18 mm. The main differences were observed in the convexity of the weld face, particularly in specimen (b), where the influence of the filler material was clearly visible. Despite these geometric differences, the obtained welds exhibited a typical LBW profile with a dendritic microstructure; however, the direction of dendrite growth differed between the analyzed specimens [30,31].

The solidification behaviour of welded materials strongly depends on their chemical composition, thermophysical properties, and welding parameters (Table 1, Table 2 and Table 3). Due to using two different welding approaches—welding with and without filler—the crystallization rates are expected to differ. To investigate this phenomenon, a WRC-1992 diagram and the G/R ratio were analyzed based on Cr_eq and Ni_eq calculations.

Weld volumes were measured using macrophotography, yielding 6.29 mm^2^ for the weld without filler and 8.28 mm^2^ for the weld with filler metal. The percentage of filler material in the weld was estimated at 10.2%. For the Cr_eq and Ni_eq calculations, an equal mass ratio (50%-50%) of both base materials was assumed. The calculated Cr_eq value for the weld without filler was 21.66, while for the weld with 309L filler it was 21.89. The Ni_eq values were 18.47 for the weld without filler and 18.1 for the weld with filler. As a result, the Cr_eq/Ni_eq ratio was 1.1727 for the weld without filler and 1.209 for the weld with 309L filler. The estimated G/R ratio for laser welding (rapid cooling) was approximately 0.6–0.7 for the weld without filler, and 0.6–0.8 for the weld with filler material. The ferrite number (FN) for the weld without filler was 2–4, while for the weld with filler it was 3–6.

### 3.2. Microstructure Analysis

**Figure 5:**
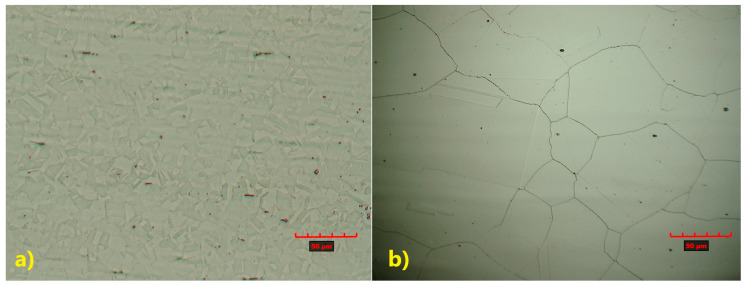
Microstructure of base materials: (**a**) TP347, (**b**) TP904L steel.

Metallographic structure analysis was performed using an optical microscope and a scanning electron microscope (SEM). Both welded stainless steels exhibit an austenitic microstructure; however, clear differences in grain size are observed (Figure 5).

TP347 steel (Figure 5a) exhibits a fine-grained austenitic microstructure, in contrast to the coarse-grained structure observed in TP904L steel (Figure 5b).

#### 3.2.1. Microstructure of Joints Welded Without Filler

The use of a laser beam as the heat source provides a high-power density, enabling high-speed welding, which influences both the weld geometry and its microstructure. In the joint welded without filler material, the heat-affected zone (HAZ) differed between the two base materials.

**Figure 6:**
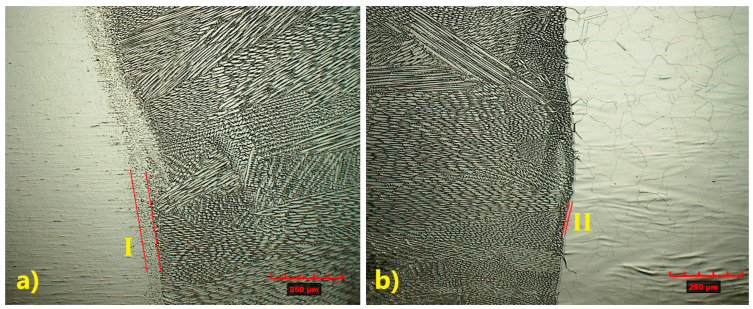
Microstructure of HAZ: (**a**) TP347 steel, (**b**) TP904L steel (×200 magnification).

Along the weld line in TP347 steel, a wider grain refinement region is observed (Figure 6—I), whereas in TP904L steel, microstructure changes in the base material occur only near the weld line (Figure 6—II). The measured HAZ width was 7.5 µm for TP904L and 84 µm for TP347.

**Figure 7:**
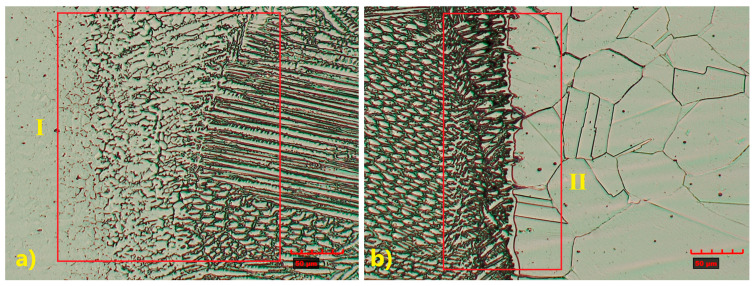
Magnified microstructure of HAZ: (**a**) TP347 steel, (**b**) TP904L steel (×800 magnification).

The microstructure of the HAZ in the TP347 steel is more complex, with evident grain refinement observed (Figure 7—I). In TP904L steel, the HAZ was difficult to precisely identify due to limited ferrite transformation, with microstructural changes occurring primarily along the fusion line (Figure 7—II) [32].

**Figure 8:**
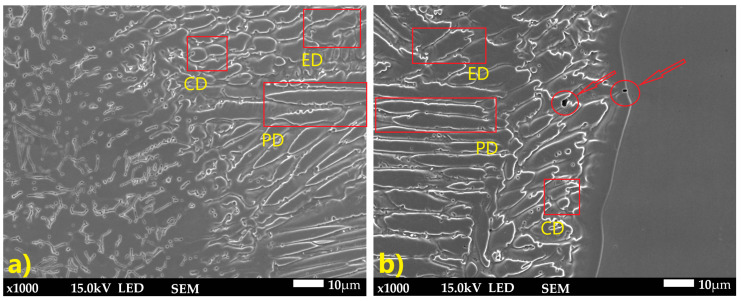
SEM microstructure of HAZ: (**a**) TP347 steel, (**b**) TP904L steel (×800 magnification).

Optical and SEM analyses (Figure 7 and Figure 8) revealed a complex weld microstructure along the fusion line, consisting of pillar (PD), cellular (CD), and equiaxed (ED) dendrites identified based on their geometry and growth orientation. In addition, several precipitates, most likely Cr_23_C_6_, caused by Cr segregation during rapid solidification, were observed in the HAZ and weld of TP904L steel near the fusion line (Figure 8b—red arrows) [18]. This side of the weld consisted primarily of pillar and equiaxed dendrites, with a small fraction of cellular dendrites. On the TP347 steel side, the weld microstructure contained all three types of dendrites; however, pillar dendrites were present in the smallest proportion (Figure 8a). The dendrites’ growth direction was perpendicular to the weld axis.

#### 3.2.2. Microstructure of Joints Welded with 309L Filler

**Figure 9:**
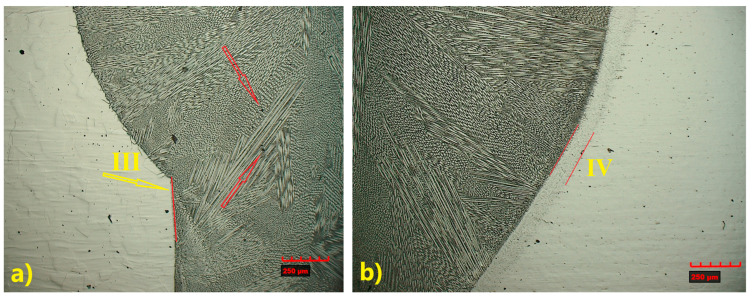
Microstructure of identified HAZ: (**a**) TP904L steel—narrow HAZ (III), (**b**) TP347 steel—wider HAZ (IV) (×200 magnification).

**Figure 10:**
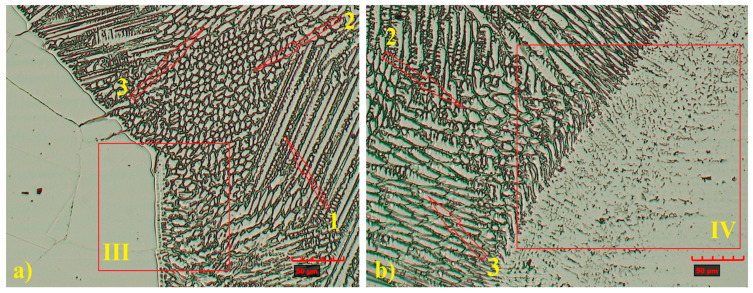
Magnified microstructure of HAZ: (**a**) TP904L steel, (**b**) TP347 steel (×800 magnification), with indicated area of dendrites types: 1—pillar, 2—cellular, 3—equiaxed.

A similar analysis was performed for the joint welded with 309L filler. Despite some observed differences in the microstructure, the weld also exhibited a typical dendritic structure, with a banded distribution of columnar and cellular dendrites (Figure 9 and Figure 10) [33].

As a result of using the same welding parameters for both joints (with and without filler material), the size and microstructure of the HAZ were similar. A wider HAZ is observed in TP347 steel (IV), measuring 106 µm (Figure 9b), whereas TP904L steel (III) exhibited a narrower HAZ of approximately 7.9 µm (Figure 9a). Precipitations (most likely Cr_23_C_6_) were observed in TP904L steel (Figure 9a—red arrows).

**Figure 11:**
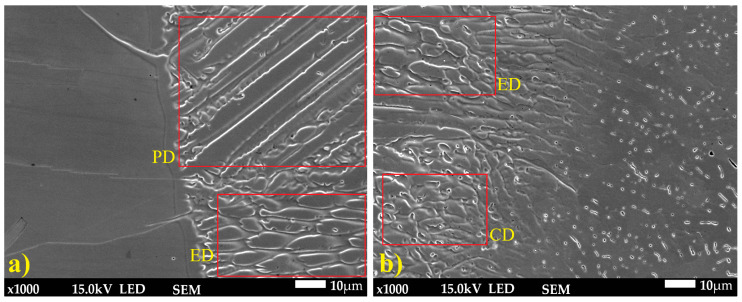
SEM microstructure of HAZ: (**a**) TP904L steel, (**b**) TP347 steel (×1000 magnification).

The weld microstructure near the fusion line consisted of cellular (region 2), equiaxed (region 3) and pillar (region 1) dendrites arranged in bands. Longer pillar dendrites are observed on the TP904L steel side (Figure 10 and Figure 11). In the joint welded with filler, a tendency for dendrite growth toward the top of the weld was observed. In the joint without filler, the dendrites’ growth direction was a combination of orientations, toward the weld axis, perpendicular to it, and toward the weld top. Magnified areas provide a detailed view of the weld microstructure (Figure 11), which corresponds well with the global observations obtained from the macrostructure images.

#### 3.2.3. Comparative Weld Microstructure and Composition Analysis

**Figure 12:**
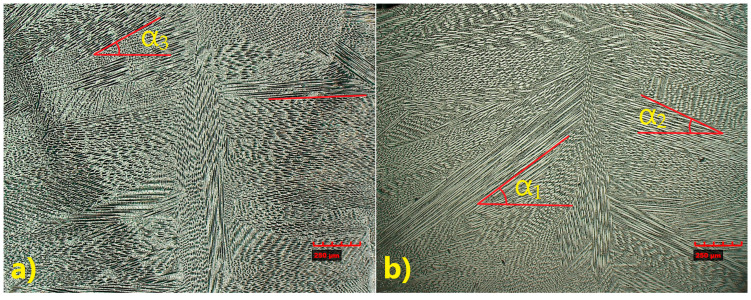
Microstructure obtained TP347—TP904L welds: (**a**) without filler materials, (**b**) with 309L filler material (×200 magnification).

Global observation and the HAZ study revealed clear differences in the microstructure of the obtained joints, suggesting the influence of 309L filler material. Therefore, a detailed study of the central area of the welds, including dendrite orientation angles, is performed (Figure 12).

The microstructure observed along the weld axis confirmed the direction of crystallization toward the top of the weld in the joint welded with filler (Figure 12b), whereas in the joint without filler, a tendency to crystallize perpendicular to the weld axis, combined with upward growth, was observed (Figure 12a). The measured growth angles of the pillar dendrites in the first weld (without filler) ranged between 0° and α_3_ = 30°, while in the second weld (with fillet metal), they were typically between α_2_ = 30° and α_1_ = 45°.

**Figure 13:**
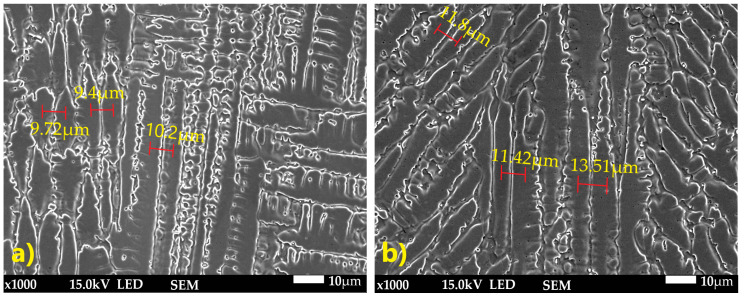
SEM image with DAS measurement: (**a**) weld without filler metal, (**b**) weld with 309L filler (×1000 magnification).

SEM microstructure analysis (Figure 13) revealed that, in general, the dendrites grew parallel to the weld axis; however, in the joint welded with 309L filler, dendrites with tilted growth orientation were observed. Dendrite arm spacing (DAS) measurements showed differences between the two joints. The average DAS for the joint without filler was 9.77 µm, whereas for the joint welded with 309L filler it was 12.25 µm.

**Figure 14:**
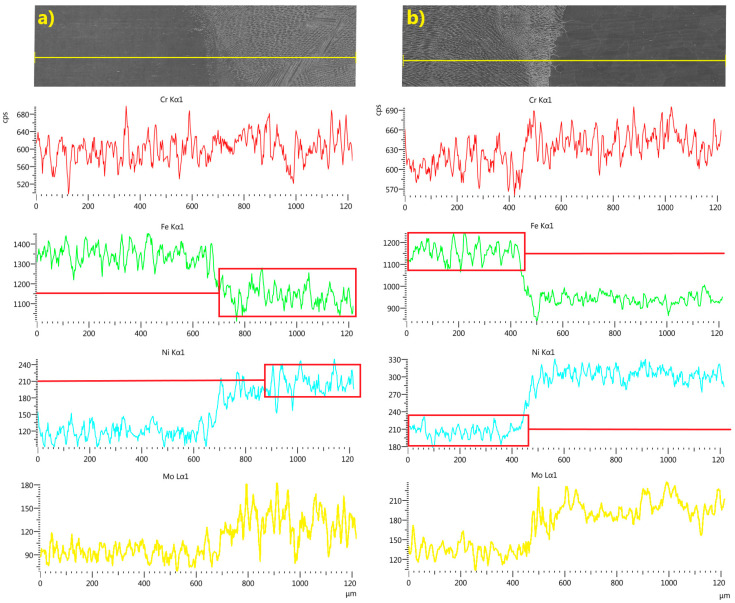
Linear EDS analyses of chromium, iron, nickel and molybdenum across the weld line in LBW joints without filler: (**a**) weld, side of TP347, (**b**) weld, side of TP904L, with graphic box/line presentation of variation and average distribution value in weld.

**Figure 15:**
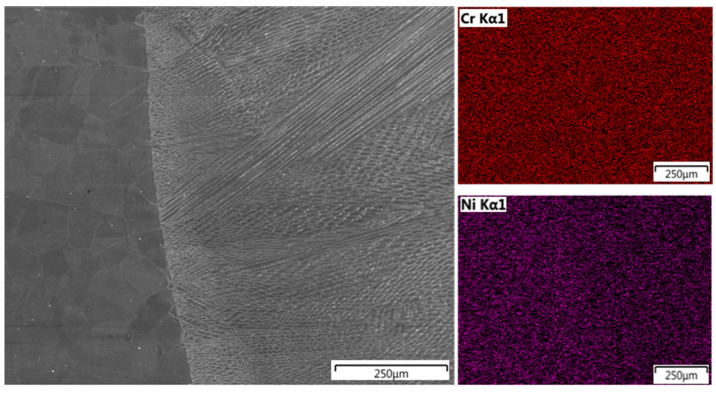
EDS map analysis of chromium and nickel distribution in TP904L—weld, joint welded without filler metal.

The composition of the weld was studied using EDS line scan and mapping analyses. The results for the joint welded without filler are shown in Figure 14 and Figure 15.

EDS line scan analysis showed a uniform distribution of chromium along the measurement line, with a slightly higher concentration in the TP904L base material. The uniform distribution of the main alloying elements—nickel, iron, and molybdenum—indicates a high mixing factor (Figure 14), which was further confirmed by chromium and nickel mapping analyses (Figure 15). The measured concentrations in the weld cross-section were within the range of the nominal composition of the base materials.

**Figure 16:**
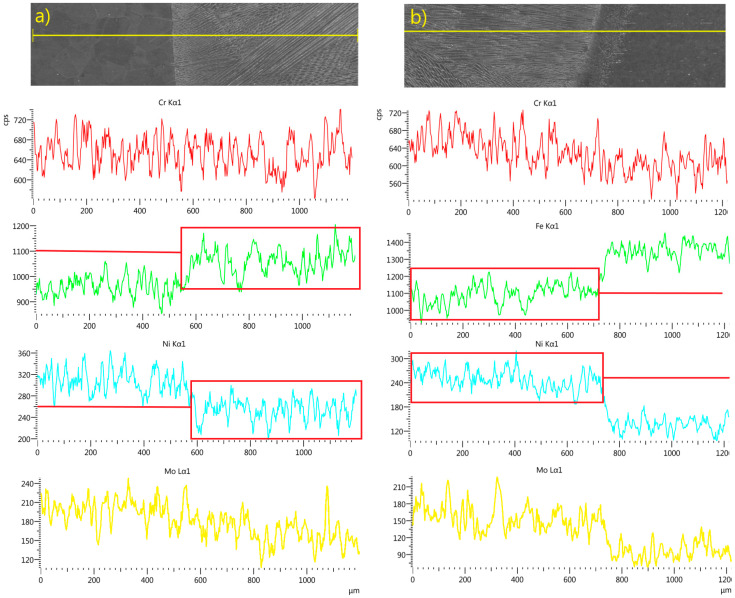
Linear EDS analyses of chromium, iron, nickel and molybdenum across the weld line in LBW joints with 309L filler: (**a**) weld, side of TP347, (**b**) weld, side of TP904L with graphic box/line presentation of variation and average distribution value in weld.

**Figure 17:**
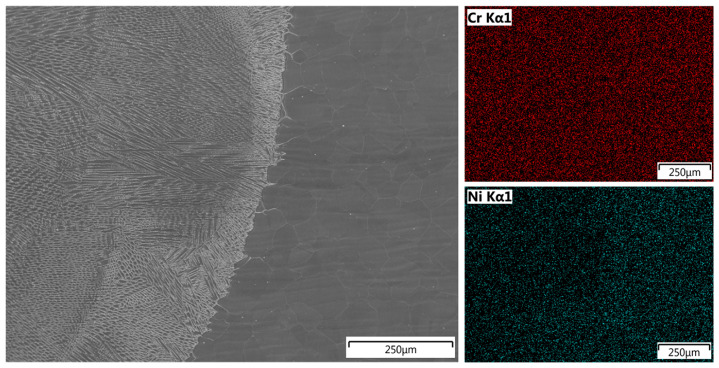
EDS map analysis of chromium and nickel distribution in TP904L—weld, joint welded with 309L filler.

Results of the joint welded with 309L filler are presented in Figure 16 and Figure 17.

The second welded joint exhibited similar results, with an increased chromium content due to the use of 309L filler wire (Table 1) and a slightly higher nickel concentration compared to the first analyzed weld (Figure 16).

Mapping analysis confirmed a uniform distribution of the analyzed elements, with a slightly higher nickel content observed in the weld area (Figure 17).

### 3.3. Mechanical Properties Analysis

**Figure 18:**
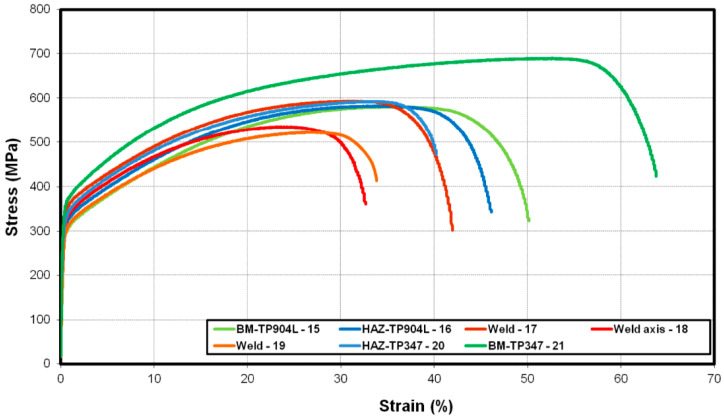
Engineering stress–strain curves of the miniaturized tensile test of the laser-welded TP904L-TP347 joint without filler.

**Figure 19:**
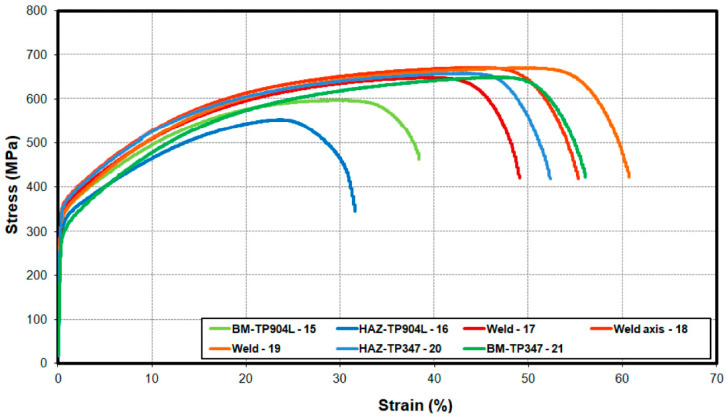
Engineering stress–strain curves of the miniaturized tensile test of the laser-welded TP904L-TP347 joint with 309L filler.

The mechanical properties of the welded joints were studied using both standard and miniaturized tensile tests. In miniaturized tensile test, the average results obtained from five specimens—with standard deviation of 14 (confidence intervals 8.42 ≤ σ ≤ 28.52 at a 95% confidence level) for specimens welded without filler, and 11 (confidence intervals 6.83 ≤ σ ≤ 26.05 at a 95% confidence level) for specimen welded with 309L filler—are presented in Figure 18 and Figure 19. Results for the base material are shown in green, the weld in red, and the HAZ in a blue colour scale.

The results of the tensile test showed the highest elongation (above 55%) and strength (up to 700 MPa) in the base material of TP347 steel (21). For TP904L (15), the corresponding values were approximately 45% elongation and 580 MPa tensile strength (Figure 18).

In HAZ, both analyzed materials exhibit similar results. In TP904L HAZ (16), elongation was 40% with a strength of 590 MPa, while in TP347 HAZ (20), the tensile strength reached 600 MPa with an elongation of 35%.

In the weld, the measured elongation ranged between 25% and 35%, with tensile strength between 520 and 580 MPa.

Greater differences were observed in the second joint, where 309L filler was used. The measured strength and elongation of the base materials TP347 and TP904L were similar to the value obtained from the first specimen—up to 700 MPa with 50% elongation for TP347 (21), and 590 MPa with 46% elongation for TP904L(15).

In the TP347 HAZ (20), elongation was 45% and tensile strength was 650 MPa, whereas in the TP904L HAZ (16) these values were 25% and 550 MPa, respectively (Figure 19). In weld, the mechanical properties improved significantly compared to the first specimen, with tensile strength values ranging from 620 to 650 MPa and elongation between 45% and 55%.

**Figure 20:**
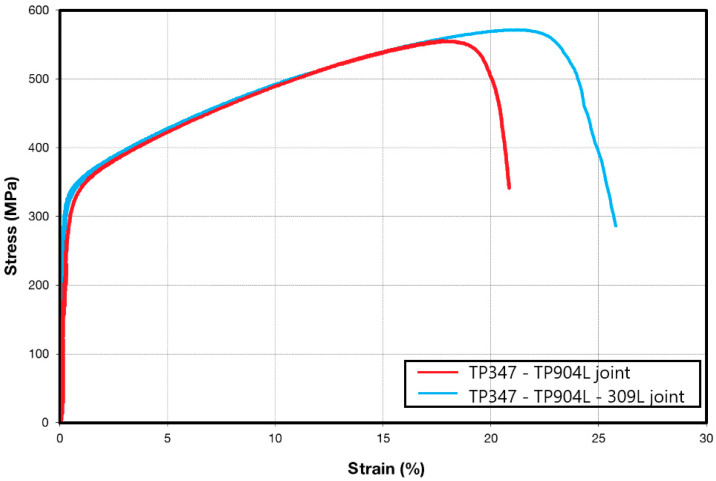
Engineering stress–strain curves for the standard tensile test of the obtained joints, welded with and without 309L filler.

The standard tensile test showed good mechanical properties of the obtained joints. Higher strength was measured in the joint welded with 309L filler—582 MPa with 23% elongation—while the joint welded without filler exhibited 550 MPa tensile strength and 18% elongation (Figure 20). The results of the standard tensile tests are consistent with the miniaturized tensile test results, confirming that the use of 309L filler improves the mechanical properties of welded joints. In both cases, a specimen fracture occurred in the TP904L base material.

**Figure 21:**
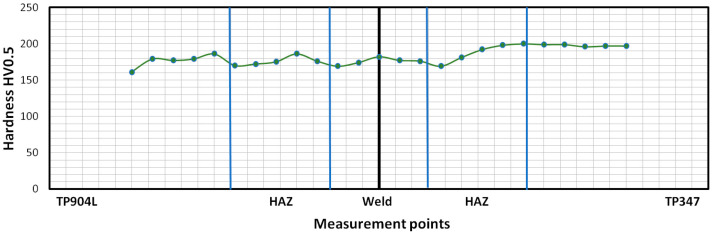
Hardness distribution of laser-welded TP904L-TP347 joints without filler material.

**Figure 22:**
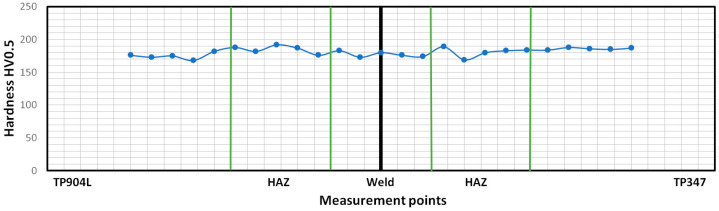
Hardness distribution of laser-welded TP904L-TP347 joints with 309L filler.

Hardness profile of the BM, HAZ and welds of the obtained joints are presented in Figure 21 and Figure 22. Vickers hardness measurement was performed along a measurement line placed at half the thickness of the weld.

The hardness test for the specimen welded without filler showed the highest hardness value in the BM and HAZ of TP347 steel, reaching 200 HV0.5, while in the weld, it slightly exceeded 180 HV0.5. Elevated hardness values were also observed in the HAZ of TP904L steel compared to the weld; however, measured values did not exceed 190 HV0.5 (Figure 21). Overall, the hardness distribution ranged from 160 HV0.5 to 200 HV0.5.

Hardness measured for specimen 2, in which 309L filler was used, showed a more uniform distribution. The highest measured value, 190 HV0.5, was observed in the HAZ of both welded materials as well as in the TP347 base material. In the weld, the hardness ranged from 170 HV0.5 to 180 HV0.5 (Figure 22). The hardness distribution was more uniform than in the first specimen, and the measured values were lower.

## 4. Discussion

Laser welding of austenitic high-alloy steels allows the preservation of joint properties comparable to those of the base materials. When an appropriate filler material is used, the properties of the welded joint can be further enhanced. In this study, 309L filler metal was selected due to its chemical composition, which is compatible with the welded materials and provides a higher tendency to ferrite formation. This composition also ensures excellent corrosion resistance. Additionally, the nominal strength of the 309L filler is higher than that of both base materials; therefore, an improvement in the mechanical properties of the welded joint was anticipated [34].

Both obtained joints exhibit proper weld build; however, the presence of filler material is clearly visible in specimen 2. The weld in this specimen is approximately 4.1 mm wider than in specimen 1, and its weld face is more convex. The differences in the weld root width between the analyzed specimens were 1.8 mm.

The welded materials exhibit a native austenitic microstructure, with TP347 showing a fine-grain structure and TP904L a coarse-grain one. The heat-affected zone (HAZ) in both welded joints was similar in nature, with its width dependent on the specific base material. A wider region of the microstructure transformation was observed in TP347 due to its higher iron and carbon contents. Moreover, rapid heating during laser welding dissolves NbC precipitates, which normally contribute to grain boundary pinning; their dissolution enables nucleation and growth of newly refined grains during rapid solidification, resulting in the observed grain refinement. In contrast, TP904L exhibited a very narrow HAZ, with no evidence of austenite grain growth. This behaviour is attributed to the high Ni content, which stabilizes the austenitic phase and limits solid-state transformation. The minimal HAZ transformation that did occur was primarily associated with δ-ferrite formation along the fusion line. Sent for revised review, elongated δ-ferrite grains formed a discontinuous network around the austenite grains. Such regions are typical in the steel with a native austenitic structure containing δ-ferrite. At high temperatures near the fusion line, a γ→δ transformation occurred, beginning in pre-existing δ-ferrite grains and in regions enriched with chromium. During cooling, these regions did not reach equilibrium, resulting in a local increase in δ-ferrite fraction. Thu, the more complex HAZ structure observed in TP347 HAZ is the combined effect of its higher carbon and iron content, along with the more pronounced ferrite formation during the welding process.

No clear differences in the HAZ microstructure were observed between the two analyzed joints. However, clear differences appeared in the weld zones. Both welds exhibit a typical dendritic microstructure consisting of pillar, cellular, and equiaxed dendrites. TP904L steel showed a tendency to form precipitates (most likely Cr_23_C_6_) (Figure 8b and Figure 9a), although their quantity was significantly lower than that reported for similar TP904L welds in the literature [22]. This reduction is attributed to mixing of alloying elements from TP904L and TP347 steels, including niobium; however, the niobium content was insufficient to fully stabilize carbide formation, resulting in the presence of some precipitates [15,18].

Differences in dendrite growth patterns indicate strong fluctuations in the weld pool flow field. Discrete growth bands or striations were observed; however, the overall structural pattern remained consistent across these bands. It is important to note that the process parameters, including the laser output power, were identical for both welds. The main differences in the weld morphology were associated with weld volume—which corresponds to the single-U or double-U profile—and the dendrite growth orientation. In the joint welded without filler metal, the dendrite growth angle did not exceed 30°, and a considerable number of dendrites were oriented perpendicular to the weld axis. In contrast, in the joint welded with filler metal, the dendrite growth angle ranged from 30° to 45°, indicating a lower thermal gradient, partly due to the laser energy being used to melt the filler wire. Additionally, the crystallization rate was reduced as a result of the filler metal’s chemical composition.

The first weld configuration (without filler) may be more susceptible to hot cracking, as suggested by the calculated ferrite number (FN = 2–4), which requires precise control of welding parameters. In contrast, the second weld (with 309L filler), with a calculated FN of 3–6, exhibited greater resistance to hot cracking and a more stable solidification structure [35,36,37]. According to the WRC-1992 diagram, AF-type (austenitic with ferrite at grain boundaries) solidification is expected in both analyzed joints.

EDS linear and map analyses confirmed a uniform distribution of alloying elements within the welds, indicating a high degree of mixing (Figure 14, Figure 15, Figure 16 and Figure 17). The chromium distribution in the joint cross-section is homogeneous, while molybdenum exhibits smoother compositional transitions in the weld produced with filler material (Figure 16) compared to the weld made without filler (Figure 14). More pronounced variations were observed in the distributions of iron and nickel; the higher iron content in TP347 compared to TP904L resulted in a stepwise transition at the weld interface. In the weld, the iron concentration was lower in the joint with 309L filler, whereas the nickel concentration was higher. This compositional shift—characterized by reduced iron and increased nickel content—enhances both corrosion resistance and mechanical performance [38].

The mechanical properties analysis revealed clear differences between the two joints. The miniaturized tensile tests of the BM, HAZ, and weld regions (Figure 1 and Figure 3) confirmed the strengthening effect produced by the use of 309L filler metal. The measured tensile strength of the weld in the joint welded with filler was up to 130 MPa higher compared to the joint without filler, and the elongation increased by up to 20% (Figure 19). The results for the base materials were comparable, while minor variations in HAZ performance were attributed to local thermal gradients and potential differences associated with specimen extraction.

The standard tensile tests further confirmed the strengthening effect; the specimen welded with 309L filler reached a tensile strength of 582 MPa, whereas the joint produced without filler achieved 550 MPa. These results demonstrate that the use of 309L filler metal in laser welding enhances the mechanical properties of the TP347–TP904L joint.

The hardness test results showed only minor variations across the joint cross-sections—approximately 40 HV0.5—reflecting the predominantly austenitic microstructure. The locally increased hardness in the TP347 HAZ was associated with δ-ferrite formation, which was consistent with the ferrite number (FN) calculations. A higher hardness in the weld metal was expected due to the finer dendrite arm spacing in the joint produced without filler, whereas the elevated hardness in the HAZ resulted from the higher thermal gradient characteristic of this welding configuration.

The obtained joints were intended for components in energy-sector installations; therefore, selected mechanical properties, particularly tensile strength, must comply with relevant standards. According to ASME Section VIII (material group referenced in Table UHA-23), the tensile strengths measured for both joints—550 MPa and 582 MPa—exceed the minimum required strength of the base materials (Table 2). Furthermore, both joints also meet the requirements of ASME B31.3, which stipulates that the tensile strength of the welded joint must exceed that of the weakest base material—TP904L steel [39,40].

## 5. Conclusions

Laser welding of high-alloy steels can produce high-quality joints, while certain properties—such as strength and corrosion resistance, which are influenced by the chemical composition of the weld—can be further enhanced through the use of a filler material. The present study demonstrates that selecting an appropriate filler metal, such as 309L, can significantly improve joint performance. The use of 309L filler increased tensile strength by 32 MPa according to standard tensile tests, and by up to 130 MPa based on miniaturized tensile test results. Additionally, weld ductility was improved by approximately 20%. Hardness measurements indicated a slight decrease in values, but with a more uniform distribution across the weld. The obtained welds exhibited proper build characteristics typical of the LBW process, with no detectable defects related to the joining procedure.

The refined weld microstructure achieved with the 309L filler also reduces susceptibility to hot cracking due to enhanced ferrite formation, as reflected by the increased ferrite number (FN). Future work, including corrosion resistance assessments and tensile testing at elevated temperatures, is planned to provide a comprehensive evaluation of the mechanical and functional properties of these welded joints.

## Figures and Tables

**Table 1:** Chemical composition of TP347 and TP904L steels and filler wire [%].

Element	TP904L	TP347	309L Filler
Fe	balanced	balanced	balanced
Cr	19.75	17.4	23.2
Ni	24.5	9.053	13.4
Mo	4.27	0.433	0.1
Mn	1.56	1.863	1.8
Si	0.51	0.312	0.4
Nb	0.0	0.464	0.01
C	0.013	0.043	0.02

**Table 2:** Mechanical and thermal properties of TP347, TP904L and 309L.

Property	TP347	TP904L	309L Filler	Unit
Tensile Strength	515–690	490–715	550–750	MPa
Yield Strength	205	220	350–420	MPa
Elongation	≥35–40	≥35	≥25–40	%
Hardness	175	170	195	HV
Elastic Modulus	193	195	200	GPa
Thermal Conductivity	16	14	14	W/m·K
Thermal Expansion Coefficient	15.8	17.2	16.5	10^−6^/K
Melting Range	1350–1390	1350–1390	1370–1420	°C
Max Service Temperature	870	500	1100	°C

**Table 3:** Laser welding parameters.

Parameter	Value	Unit
Laser output power	5.5	kW
Frequency	20	kHz
Welding speed	1	m/min
Wire feed rate	1.2	m/min
Shielding gas flow (helium)	15	L/min

## Data Availability

The original contributions presented in this study are included in the article. Further inquiries can be directed to the corresponding author.
